# Omalizumab for the Treatment of Chronic Spontaneous Urticaria in Adults and Adolescents: An Eight-Year Real-Life Experience

**DOI:** 10.3390/jcm13185610

**Published:** 2024-09-21

**Authors:** Paolo Calzari, Alessandra Chiei Gallo, Francesca Barei, Eleonora Bono, Massimo Cugno, Angelo Valerio Marzano, Silvia Mariel Ferrucci

**Affiliations:** 1 Dermatology Unit, Fondazione IRCCS Ca’ Granda Ospedale Maggiore Policlinico, 20122 Milan, Italy; paolo.calzari@unimi.it (P.C.); alessandra.chiei@unimi.it (A.C.G.); angelo.marzano@unimi.it (A.V.M.); 2 Department of Pathophysiology and Transplantation, Università Degli Studi di Milano, 20122 Milan, Italy; eleonora.bono@unimi.it (E.B.); massimo.cugno@unimi.it (M.C.); 3 Fondazione IRCCS Ca’ Granda Ospedale Maggiore Policlinico, SC Medicina—Emostasi e Trombosi, 20122 Milan, Italy

**Keywords:** chronic spontaneous urticaria, omalizumab, drug survival

## Abstract

**Background**: Omalizumab, an anti-IgE monoclonal antibody, is an effective treatment for patients with chronic spontaneous urticaria (CSU) resistant to antihistamines, but about 10% are unresponsive. Our aim was to assess the effectiveness, safety, and drug survival (DS) of omalizumab by considering clinical and laboratory characteristics. **Methods**: We conducted a retrospective study on 296 patients with severe CSU treated with omalizumab. Disease activity, comorbidities, and serum levels of total IgE and anti-thyroid autoantibodies were evaluated over a period of up to 8 years. DS was analyzed using unadjusted Kaplan–Meier survival curves. When applicable, the risk of discontinuation was assessed using Cox regression analysis. **Results**: Out of 296 patients, 118 (40.4%) were early responders, 72 (25.0%) were late responders, 76 (26.0%) were partial responders, and 25 (8.6%) were non-responders. Early responders were more likely to be patients without associated inducible urticaria (*p* = 0.021, χ^2^ = 9.692), without autoimmune thyroiditis (*p* = 0.007, χ^2^ = 12.037), and those with higher IgE levels (*p* = 0.039, χ^2^ = 8.385). Overall, DS was 53.5% at 8 years, primarily due to clinical remission. DS due to inefficacy and clinical remission were 83.9% and 62.1%, respectively, at 8 years. No patients discontinued omalizumab due to adverse events. Patients with normal IgE levels (*p* = 0.012, HR = 4.639, CI: 1.393–15.445) and those with autoimmune thyroiditis (*p* = 0.028, HR = 3.316, CI: 1.128–8.718) had a higher risk of discontinuing omalizumab due to inefficacy. **Conclusions**: This study confirms the long-term effectiveness and safety of omalizumab in the treatment of CSU over a period of up to 8 years. Most patients discontinued omalizumab due to clinical remission, while only 5.1% discontinued it due to ineffectiveness.

## 1. Introduction

Chronic spontaneous urticaria (CSU) is a clinical condition characterized by the spontaneous appearance of short-lived wheals (<24 h), angioedema, or both, lasting for more than six weeks [1]. By definition, the symptoms occur without any specific trigger; however, factors such as infections, NSAIDs, stress, and other aggravators can increase disease activity [1,2,3,4]. CSU typically persists for 2–5 years before resolving on its own [5,6]; however, in up to 30% of patients, it can last for many years or even decades [4,6,7]. During this time, patients struggle with pruritus, the most impactful symptom of CSU, which affects their quality of life by causing sleep disturbances, reduced physical and emotional well-being, and decreased performance at school and work [8,9]. Patients have also been shown to experience higher rates of psychiatric comorbidities, such as anxiety, depression, and somatoform disorders, compared to controls [10].

Globally, the estimated point prevalence of CSU ranges from approximately 0.5% to 1%, with a notable female predominance (female-to-male ratio of 2:1) [11]. These data highlight the significant impact of the condition on both patients and society [4].

Treating CSU is challenging. Current international guidelines recommend a three-tiered approach: start with second-generation H1 antihistamines at approved dosages, then escalate the dosage up to four times if necessary, and finally introduce omalizumab, the first anti-immunoglobulin (IgE) monoclonal antibody [12]. While many patients with CSU achieve complete or substantial control of their symptoms with the standard treatment approach, some remain unresponsive. In such cases, it becomes necessary to switch to immunosuppressive treatments such as cyclosporine or azathioprine, as recommended by the European Guidelines [1]. The response to omalizumab varies, ranging from an early response (ER) to no response (NR) at all, with intermediate cases of late or partial response, indicating that different underlying mechanisms could be involved [13]. Early responders likely have an IgE-mediated autoallergic disease (type I CSU) involving a range of autoallergens that trigger mast-cell-bound autoimmune IgE [14]. In contrast, late responders or non-responders typically have an IgG-mediated autoimmune disease (type IIb CSU), where IgG targets IgE or its high-affinity receptor [15,16,17,18]. Both pathways can ultimately cause mast cell degranulation and histamine release.

Studying the pathogenesis of CSU has led to the development of the concept of disease-modifying treatments (DMTs). Defined as interventions that not only alleviate symptoms but also fundamentally alter the underlying mechanisms driving the disease, DMTs are designed to prevent or delay disease progression, achieve long-lasting remission without ongoing therapy, and directly impact the underlying disease mechanisms. Examples include reducing the production of autoantibodies or targeting specific cytokines that contribute to inflammation and symptom manifestation [19]. Effective drugs include omalizumab, dupilumab—a humanized IgG4 monoclonal antibody targeting the IL-4 and IL-13 receptor α-chain, remibrutinib—an inhibitor of Bruton’s tyrosine kinase (BTK), and other promising agents currently under study for CSU [19].

The objective of this study is to analyze eight years of real-life data on omalizumab use in chronic spontaneous urticaria (CSU). We aim to gather baseline data and assess the efficacy and safety of omalizumab, considering early responders, late responders, partial responders, and non-responders. Additionally, we conducted an analysis of omalizumab drug survival. The latter reflects routine clinical practice by measuring the duration from therapy and initiation to discontinuation. Drug survival represents a comprehensive indicator of both effectiveness and safety.

## 2. Patients and Methods

### 2.1. Study Population

We conducted a retrospective study from 2016 (when the first case received omalizumab) until 2024 (data lock). We studied 296 patients with severe CSU unresponsive to antihistamines and treated with omalizumab. All clinical evaluations were performed at the Department of Dermatology, Foundation IRCCS, Ca’ Granda Ospedale Maggiore Policlinico, Milan, Italy. The inclusion criteria, in accordance with current Italian legislation, were (1) a baseline UAS-7 score greater than 16 and (2) administration of a fixed dose of 300 mg of omalizumab subcutaneously every four weeks. The exclusion criteria were (1) neoplastic disease or (2) pregnancy. 

At baseline, the following characteristics were recorded: sex, age, age at CSU onset, presence of angioedema, inducible urticaria, atopic comorbidity, presence of Hashimoto’s thyroiditis, dermatological comorbidities, other comorbidities, and previous antihistamines therapies. The severity of CSU was assessed using the Weekly Urticaria Activity Score (UAS-7). This score is based on the patient’s assessment of key urticaria signs and symptoms, specifically wheals and pruritus. Patients document these signs and symptoms as none, mild, moderate, or intense, corresponding to scores of 0, 1, 2, and 3, respectively. The scores are recorded daily and summed over seven days, resulting in a total score that ranges from 0 to 42. Thyroid autoimmunity was determined based on a positive anti-TPO assay, regardless of thyroid function status. A result exceeding 60 IU/mL was classified as positive. Given that the cutoff for IgE varies by laboratory, we dichotomized IgE levels into normal levels and high levels based on the cutoff of the individual laboratory. The response to omalizumab was evaluated according to the reduction in UAS-7 as follows: early response (ER) is characterized by a disappearance or a reduction of more than 80% in the UAS-7 score within 4 weeks of the first dose. A late response (LR) is defined as a reduction of more than 50% in the UAS-7 score within 1 to 3 months after the first dose. Patients who experienced a reduction in more than 50% in the UAS-7 score with omalizumab but showed no further improvement were classified as partial responders (PR). Non-response (NR) is defined as the absence of any improvement, specifically a failure to achieve at least a 50% reduction in UAS-7 scores after 3 months of treatment [13].

Adverse events were graded according to the Common Terminology Criteria for Adverse Events (CTCAE—version 5.0, 2017) as mild, moderate, severe, life-threatening, or resulting in death.

Written informed consent was obtained from the patients or parents of minors to publish their data in an anonymous form. The internal review board approved the study in April 2022. Since the study was exclusively observational and based solely on routine analysis, formal approval by an external ethical committee was not warranted or requested. The study was conducted by following the ethical principles of the 2013 revision of the Declaration of Helsinki and the code of Good Clinical Practice.

### 2.2. Laboratory Methods

Anti-TPO IgG autoantibodies and total IgE levels were measured using commercial enzyme fluoroimmunoassays, specifically EliA, anti-TPO, and EliA total IgE from Thermo Fisher Scientific (Waltham, MA, USA).

### 2.3. Statistical Analysis

Descriptive statistics are reported as mean and standard deviation (SD) or median and 25–75° quartile (Q1–Q3) for quantitative variables based on the distribution of the population. Absolute numbers (*n*) and frequencies (%) are used for categorical variables. Dichotomous non-normal distributions were tested using the Mann–Whitney U test. Categorical variables were analyzed using a chi-square test or Fisher’s exact test, as appropriate. Drug survival was analyzed using unadjusted Kaplan–Meier survival curves to estimate the risk and time to discontinuation. Patients were censored if, at the July 2024 data lock, they were still under treatment with omalizumab, lost to follow-up, or discontinued due to causes not related to omalizumab or clinical remission. The log-rank test was used to assess differences in drug survival across baseline epidemiological and clinical characteristic groups. We considered the investigation of time-dependence in Cox’s proportional hazards model using the graphical analysis (a log-minus-log plot) and Schoenfeld residuals. The data were analysed using the SPSS PC statistical package, version 29 (IBM SPSS Inc., Chicago, IL, USA).

## 3. Results

### 3.1. Study Population

Out of 296 CSU patients, 192 (64.9%) were female and 104 (35.1%) were male. Among these, 290 patients were ≥18 years old (98.0%) and 6 were <18 years old (2.0%) (Table 1). According to WHO, adolescence is defined as ages 10 to 19. In our case list, five patients were 19 years old, four were 18, two were 17, one was 16, two were 15, and one was 14 years old. 

Angioedema was present in 100 patients (33.8%), and a physical inducible component was observed in 66 patients (22.3%). The majority of patients (151, 51.0%) did not have any atopic comorbidities. Hashimoto’s thyroiditis was present in 17.6% of the patients. Elevated immunoglobulin E (IgE) levels were found in 41.6% of patients. Most patients had previously received bilastine (70.8%), followed by ebastine (39.4%), and cetirizine (30.5%). The median (Q1–Q3) UAS7 score at baseline was 22.0 (22.0–28.0). 

Women had a longer history of CSU before starting omalizumab compared to men (*p* = 0.013). Additionally, they showed a higher prevalence of Hashimoto’s thyroiditis (*p* < 0.001, χ^2^ = 20.419) (Supplementary Table S1).

### 3.2. Clinical Response to Omalizumab

In our study, 118 patients (40.4%) were early responders, 72 (25.0%) were late responders, 76 (26.0%) were partial responders, and 25 (8.6%) were non-responders. Four patients had only a 1 month follow-up, so their clinical response was not assessable. No differences in clinical response categories were found with respect to sex, age at onset, presence of angioedema, or presence of atopic comorbidities. A statistically significant difference was observed regarding the presence of inducible urticaria (*p* = 0.021, χ^2^ = 9.692). Patients without inducible urticaria were more frequently early responders, while patients with inducible urticaria were more frequently late responders (Table 2).

A statistically significant difference was found regarding the presence of Hashimoto’s thyroiditis (*p* = 0.007, χ^2^ = 12.037). Patients without autoimmune thyroiditis had a higher proportion of non-responders compared to those with thyroiditis. Conversely, patients without thyroiditis were more likely to be early responders (Table 3).

A statistically significant difference was found in relation to IgE levels (*p* = 0.039, χ^2^ = 8.385). Patients with higher IgE levels were predominantly early responders, while those with normal IgE levels had a higher percentage of non-responders (Table 4).

### 3.3. Drug Survival

In our cohort, the median observation period was 22.0 months [IQR: 9.0–45.0]. As of May 2024, 170 patients were still under active treatment, 48 were lost to follow-up, and 77 had discontinued the drug. The reasons for discontinuation included inefficacy in 15 patients, clinical remission in 58, patient decision in 4, and pregnancy in 1.

Overall drug survival rates were 95.8% at 4 months, 91.2% at 8 months, 86.6% at 12 months, 76.6% at 24 months, 72.8% at 36 months, 68.7% at 48 months, 66.4% at 60 months, 59.0% at 72 months, and 53.5% at both 84 and 96 months.

**Figure 1:**
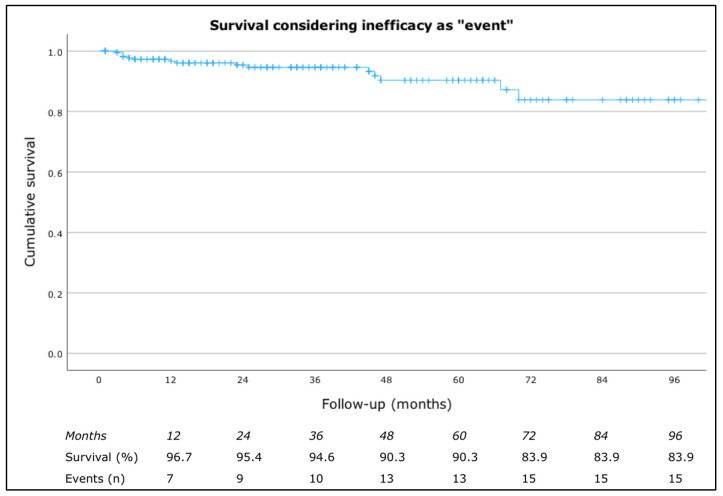
Drug survival considering inefficacy as “event”.

When considering drug survival due to inefficacy, the rates were 98.2% at 4 months, 97.2% at 8 months, 96.7% at 12 months, 95.4% at 24 months, 94.6% at 36 months, 90.3% at both 48 and 60 months, and 83.9% at 72, 84, and 96 months (Figure 1).

**Figure 2:**
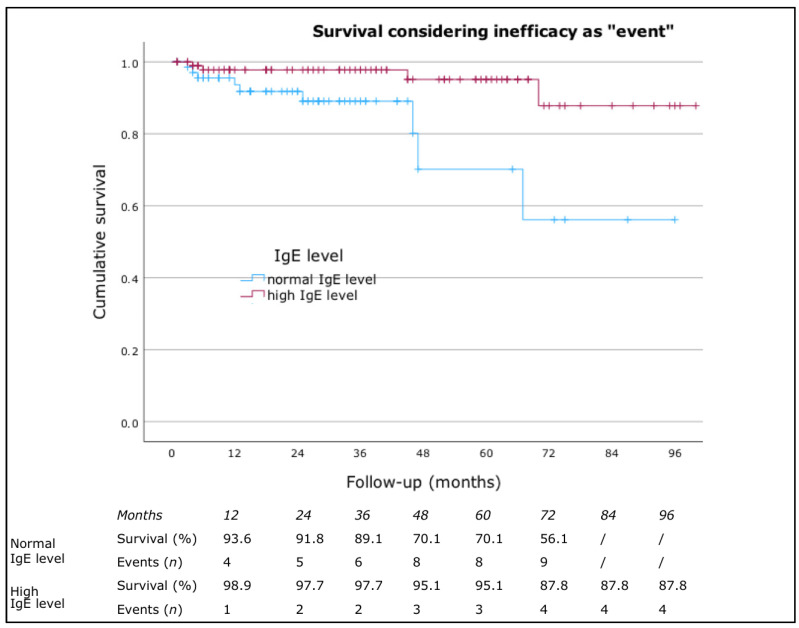
Drug survival considering inefficacy as “event” classified by IgE level. IgE, immunoglobulin E.

**Figure 3:**
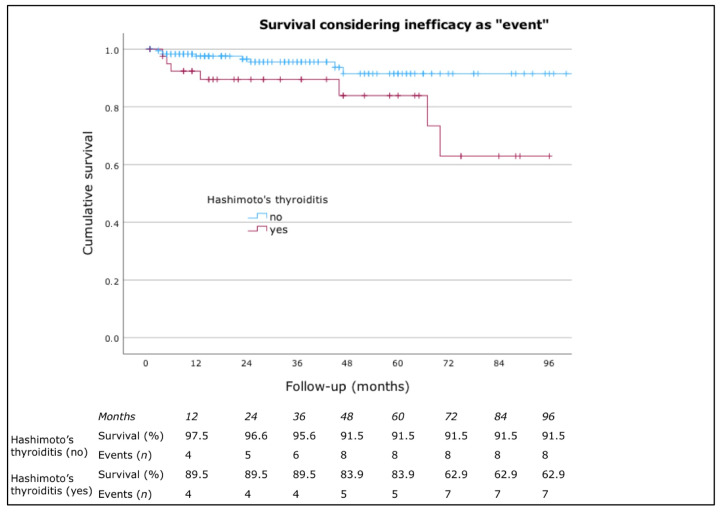
Drug survival considering inefficacy as “event” classified by the presence of Hashimoto’s thyroiditis.

No significant differences were found in drug survival based on sex, presence of atopic comorbidities, presence of angioedema, or inducible urticaria. However, a statistically significant difference was observed for Hashimoto’s thyroiditis and IgE levels. Patients with high IgE levels had a higher drug survival rate (*p* = 0.007, log-rank = 7.406) (Figure 2). According to Cox’s regression analysis, patients with normal IgE levels had a higher risk of discontinuing omalizumab due to inefficacy (*p* = 0.012, HR = 4.639, CI: 1.393–15.445). Patients without Hashimoto’s thyroiditis had a higher drug survival rate (*p* = 0.021, log-rank = 5.333) (Figure 3). Cox’s regression analysis also indicated that patients with Hashimoto’s thyroiditis had a higher risk of discontinuing omalizumab due to inefficacy (*p* = 0.028, HR = 3.316, CI: 1.128–8.718).

**Figure 4:**
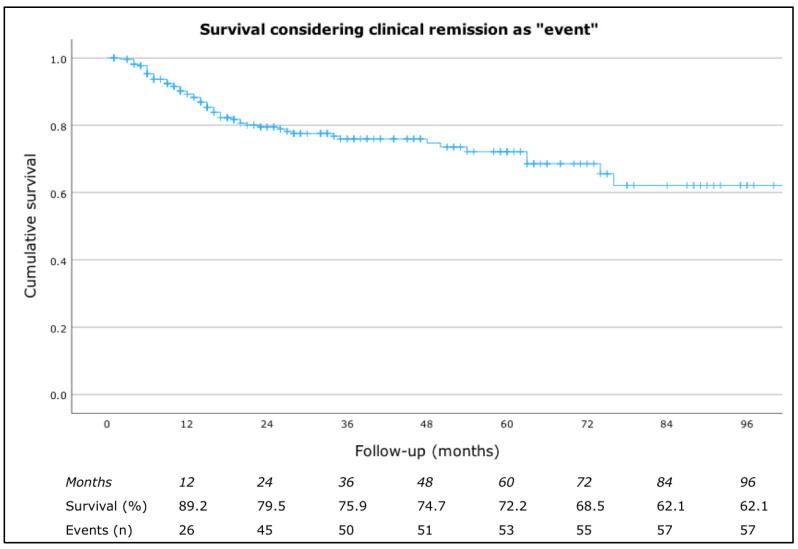
Drug survival considering clinical remission as “event”.

When considering drug survival due to clinical remission, the rates were 98.1% at 4 months, 93.7% at 8 months, 89.2% at 12 months, 79.5% at 24 months, 75.9% at 36 months, 74.7% at 48 months, 72.2% at 60 months, 68.5% at 72 months, and 62.1% at both 84 and 96 months (Figure 4). No significant differences were found with respect to sex or the presence of atopic comorbidities, angioedema, or inducible urticaria, Hashimoto’s thyroiditis, or IgE levels.

### 3.4. Effectiveness in Adolescent

Our population consisted of 15 adolescent patients, of which 11 (73.3%) were female and 4 (26.7%) were male. Three patients (20.0%) had concomitant angioedema, five (33.3%) had a physical inducible component, six (40.0%) had atopic comorbidity, and none had thyroiditis. Ten patients (66.7%) had high IgE levels. Concerning clinical effectiveness, one (6.7%) was NR, six (40.0%) were ER, two (13.3%) were LR, and six (40.0%) were PR. Two patients discontinued the drug due to clinical remission after 7 and 9 months of treatment, respectively. 

### 3.5. Safety

According to the CTCAE criteria, no patients experienced adverse events, including mild ones. Only four patients reported mild erythema at the injection site.

## 4. Discussion

This study, the first drug survival analysis of omalizumab in the Italian population, demonstrated a good clinical response and longer drug survival compared to previous studies. 

Omalizumab works by binding to free IgE, thereby reducing its levels and leading to decreased expression of the IgE high-affinity receptor, FcεRI, on basophils and mast cells. This reduction makes the cells less susceptible to activation by IgE and IgG anti-FcεRI, thereby preventing the release of histamine and reducing inflammation [12]. Omalizumab is indicated for patients who are resistant to high doses of second-generation antihistamines. While the majority of patients respond well to omalizumab, approximately 10% may remain resistant. 

In our case list, there were no significant differences in response categories based on sex, age of onset, baseline age, presence of angioedema, or atopic comorbidities. Women exhibited a longer duration of CSU before starting omalizumab; this finding could be due to differences in perception and tolerance between the sexes, as well as cultural differences. A statistical reduction in omalizumab response was observed in patients with inducible urticaria, Hashimoto’s thyroiditis, and low IgE levels. The frequency of Hashimoto’s thyroiditis was more than six times higher in women with CSU. Numerous studies indicate that IgG antithyroid autoantibodies likely do not have a direct causative or pathogenic role in CSU but may be a concurrent autoimmune occurrence [20].

Compared to the study of Asero et al. [13], which reported 52% of patients as early responders (ER) and 16% as partial responders (PR), our study observed a lower percentage of ER (40.4%) and a higher percentage of PR (26%). Additionally, Asero et al. documented a higher percentage of non-responders (NR) at 10%, compared to our 8.6%, indicating a generally favorable outcome in our cohort.

Our findings corroborate the literature that autoimmune diseases, particularly type IIb autoimmune CSU, and inducible urticaria are associated with a less favorable response to treatment [21,22]. Specifically, we found that patients with inducible urticaria were more frequently late responders (LR). Kolkhir et al. [20] also noted an association between autoimmune diseases and a reduced response to omalizumab, which our study supports, showing a higher percentage of NR among patients with Hashimoto’s thyroiditis.

The prevalence of thyroid autoimmunity in our cohort was 17.6%, comparable to Soegiharto et al. (18%) but lower than Asero et al. (24%) [13,23]. Higher baseline IgE levels have been associated with better responses to omalizumab, as described by Asero et al. and Weller et al. [13,24]. Our data align with this observation, as patients with elevated IgE levels were predominantly ER.

Furthermore, atopic comorbidity was observed in 44% of our patients, closely aligning with the findings of a recent study by Qiquan et al. in China, which reported an atopic comorbidity rate of 48% [25].

Our study reported an overall drug survival rate of 95.8% at 4 months and 86.6% at 12 months, with a gradual decline to 53.5% at 84 and 96 months. The most common reason for discontinuation in our cohort was complete clinical remission, consistent with previous studies [23,26,27]. In contrast, discontinuation due to treatment failure was rare after 1 year, suggesting that secondary resistance to omalizumab is uncommon [26]. Another study also identified the well-controlled disease as the primary reason for discontinuation; however, patients with chronic inducible urticaria (CindU) were less likely to discontinue omalizumab despite achieving well-controlled disease activity [27]. Adverse events were mild and uncommon, consistent with the existing literature by Saini et al. [28]. Four patients reported injection-site reactions; however, none discontinued omalizumab. Our data show an excellent safety profile for omalizumab, with no cases of anaphylaxis or treatment discontinuation due to adverse events.

### Limitations

The main limitation of this study is its retrospective nature. Additionally, being a single-center study poses another limitation.

## 5. Conclusions

This paper, the first drug survival study of the Italian population, confirms omalizumab’s long-term effectiveness in chronic urticaria, demonstrating long-term drug survival rates that decrease from 95.8% to 53.5% over eight years, primarily due to well-controlled disease. Discontinuation due to ineffectiveness was rare and occurred primarily early in treatment, with no discontinuations due to adverse events. Omalizumab’s efficacy and safety in this Italian cohort are consistent with the existing literature, with robust early and late response rates and long-term use improving patients’ quality of life.

## Figures and Tables

**Table 1:** Baseline clinical and epidemiological characteristics of the population study. IgE, immunoglobulin E; CSU, chronic spontaneous urticaria.

Overall Population (*n*)	296
Sex, *n* (%)	
- Male	104 (35.1)
- Female	192 (64.9)
CSU onset age, mean (SD)	42.7 (17.0)
- ≥18-year-old, *n* (%)	291 (97.2)
- <18-year-old, *n* (%)	8 (2.8)
Baseline age, mean (SD)	46.9 (16.5)
- ≥18-year-old, *n* (%)	290 (98.0)
- <18-year-old, *n* (%)	6 (2.0)
CSU duration before starting omalizumab, median (Q1–Q3)	24.1 (9.7–68.4)
Angioedema, *n* (%)	100 (33.8)
Inducible urticaria, *n* (%)	66 (22.3)
*missing*	*23 (7.8)*
Atopic comorbidities, *n* (%)	130 (43.9)
*missing*	*15 (5.1)*
Hashimoto thyroiditis, *n* (%)	52 (17.6)
*missing*	*13 (4.4)*
Dermatological disease, *n* (%)	
- Vitiligo	2 (0.6)
- Prurigo nodularis	1 (0.3)
Other comorbidities, *n* (%)	
- Rheumatoid arthritis	1 (0.3)
- Familiar colon polyposis	1 (0.3)
- Coeliac disease	1 (0.3)
- Diverticulosis	1 (0.3)
- Cardiovascular disease	1 (0.3)
- Psychiatric disease	3 (0.9)
- Familiar Mediterranean Fever	1 (0.3)
- Autoimmune atrophic gastritis	1 (0.3)
- HBV	1 (0.3)
- HCV	1 (0.3)
- Hypertension	1 (0.3)
- Lupus	3 (0.9)
- Multiple myeloma	1 (0.3)
- Sacroiliitis HLA B27+	1 (0.3)
- Sjogren syndrome	1 (0.3)
- Chronic pyelonephritis	1 (0.3)
IgE level, *n* (%)	
- High IgE level	123 (41.6)
- Normal IgE level	93 (31.4)
*missing*	*8 (27.0)*
Previous therapies, *n* (%)	
- Bilastine	167 (56.4)
- Ebastine	93 (31.4)
- Levocetirizine	36 (12.2)
- Cetirizine	72 (24.3)
- Rupatadine	47 (15.8)
- Fexofenadine	19 (6.4)
- Idroxizine	11 (3.7)
- Oxatomide	2 (0.7)
UAS7 at baseline, median (Q1–Q3)	22.0 (22.0–28.0)

**Table 2:** Distribution in number (*n*) and percentage (%) of patients with inducible urticaria by clinical response subgroups. NR, non-responder; ER, early responder; LR, late responder; PR, partial responder.

	Clinical Response Categories				
Inducible Urticaria	NR	ER	LR	PR	Total
No, *n* (%)	20 (9.9)	90 (45.8)	40 (19.7)	50 (24.6)	200 (100.0)
Yes, *n* (%)	5 (7.6)	19 (28.8)	24 (36.4)	18 (27.3)	66 (100.0)
*p* = 0.021, χ^2^ = 9.692					

**Table 3:** Distribution in number (*n*) and percentage (%) of patients with Hashimoto’s thyroiditis by clinical response subgroups. NR, non-responder; ER, early responder; LR, late responder; PR, partial responder.

	Clinical Response Categories				
Hashimoto’s Thyroiditis	NR	ER	LR	PR	Total
No, *n* (%)	15 (6.6)	98 (43.0)	58 (25.4)	57 (25.0)	228 (100.0)
Yes, *n* (%)	10 (19.6)	16 (31.4)	8 (15.7)	17 (33.3)	51 (100.0)
*p* = 0.007, χ^2^ = 12.037					

**Table 4:** Distribution in number (*n*) and percentage (%) of patients with Hashimoto’s thyroiditis by clinical response subgroups. NR, non-responder; ER, early responder; LR, late responder; PR, partial responder; IgE, immunoglobulin E.

	Clinical Response Categories				
IgE Level	NR	ER	LR	PR	Total
Normal level, *n* (%)	12 (13.0)	29 (31.5)	30 (32.6)	21 (22.8)	92 (100.0)
High level, *n* (%)	7 (5.8)	57 (47.1)	27 (22.3)	30 (24.8)	121 (100.0)
*p* = 0.039, χ^2^ = 8.385					

## Data Availability

The data presented in this study are available from the corresponding author upon reasonable request.

## Supplementary Materials

The following supporting information can be downloaded at: https://www.mdpi.com/article/10.3390/jcm13185610/s1, Table S1: Sex differences in baseline clinical characteristics of the population study. IgE, immunoglobulin E; CSU, chronic spontaneous urticaria.
