# Supplementary material for: Omalizumab for the Treatment of Chronic Spontaneous Urticaria in Adults and Adolescents: An Eight-Year Real-Life Experience
Source: J Clin Med. 2024 Sep 21;13(18):5610. doi: 10.3390/jcm13185610 (PMC11432798; doi:10.3390/jcm13185610)
Supplement: Supplementary file 1 [file jcm-13-05610-s001.zip › jcm-3174186-supplementary.pdf]

Supplementary Table S1. Sex differences in baseline clinical characteristics of the population study. IgE, immunoglobulin E; CSU, chronic spontaneous urticaria.

**Overall population (*n*)**	**Female (n=192)**	**Male (n= 104)**	**p-value**
CSU onset age, mean (SD) years<br>- Adults (≥ 18-year-old), *n* (%)<br>- Children and adolescents (<18-year-old), *n* (%)	43.2 (17.9)<br>15 (7.9)<br>177 (92.1)	41.83 (15.4)<br>8 (7.7)<br>96 (92.3)	0.667
Baseline age, mean (SD) years<br>- Adults (≥ 18-year-old), *n* (%)<br>- Adolescents (13-17-year-old), *n* (%)	47.8 (17.1)<br>187 (97.4)<br>5 (2.6)	45.3 (15.1)<br>103 (99.0)<br>1 (1.0)	0.667
CSU duration in months before starting omalizumab, median (Q1-Q3)	26.4 (10.6-80.5)	17.4 (7.3-49.0)	**0.013**
Angioedema, *n* (%)	70/192 (36.5)	30/104 (28.8)	0.186
Inducible urticaria, *n* (%)	40/175 (20.8)	26/98 (25.0)	0.496
Atopic comorbidities, *n* (%)	88/184 (45.8)	47/100 (45.2)	0.894
Hashimoto thyroiditis, *n* (%)	48/185 (25.0)	4/98 (3.8)	**< 0.001 (χ^2=20.419)**
IgE level, *n* (%)<br>- High IgE level<br>- Normal IgE level	 80/142 (41.7)<br>62/142 (32.3)	 31/74 (29.8)<br>43/74 (41.3)	0.803
